# Balancing acts: exploring work family conflict, psychological flexibility and job performance among Chinese pharmacists

**DOI:** 10.1080/20523211.2025.2450597

**Published:** 2025-02-24

**Authors:** Rabia Mahmood, Umbreen Khizar, Maham Imtiaz, Humara Adnan, Tanzila Rehman, Engidaw Abriham Ebabu, Abida Naz, Ali Ahmed Al-Halani

**Affiliations:** a Department of Chemistry, University of Education, Vehari Campus, Lahore, Pakistan; b Department of Psychology, Institute of Southern Punjab, Multan, Pakistan; c Department of Applied Psychology, The Women University Multan, Lahore, Pakistan; d Department of Mathematics, Comsats University, Islamabad, Pakistan; e Department of Chemistry, The Women University Multan, Lahore, Pakistan; f Shenzhen Research Institute, University of International Business and Economics, Shenzhen, People’s Republic of China; g Computer Science and Engineering Department, Central South University, Changsha, People’s Republic of China; h Biology Department, Faculty of Education and Applied Sciences, Hajjah University, Hajjah, Yemen

**Keywords:** Work Family conflict, psychological flexibility, job performance, pharmacists, China, healthcare

## Abstract

**Background:**

Pharmacists are key to China's healthcare system, balancing traditional Chinese medicine (TCM) and Western pharmaceuticals. The expanding pharmaceutical industry has increased their workload, contributing to work family conflict, which affects job satisfaction and performance and can lead to burnout. Psychological flexibility may alleviate the negative effects of work family conflict on job performance.

**Method:**

This study used a cross-sectional quantitative approach to examine the relationships between work family conflict, psychological flexibility and job performance among 1,359 pharmacists in Shenzhen, China. Online questionnaires assessed work family conflict using the Work and Family Conflict Scale, psychological flexibility using the Acceptance and Action Questionnaire-2 and job performance using the Individual Work Performance Questionnaire.

**Results:**

Correlation analyses revealed significant positive associations between work family conflict and psychological flexibility and between psychological flexibility and job performance. Regression analyses showed that work family conflict negatively predicted job performance, while psychological flexibility positively predicted it. Pharmacists in metropolitan areas reported higher psychological flexibility than those in rural areas, but there were no significant differences in job performance. Male pharmacists had higher psychological flexibility and job performance scores than females although the differences were not statistically significant.

**Conclusion:**

This study underlines the importance of psychological flexibility in enhancing job performance amid work family conflict. The study suggests implementing stress reduction programmes, mindfulness training and workplace policies such as flexible working hours and childcare services to reduce work family conflict and foster psychological flexibility. Addressing these issues can significantly improve the well-being and job performance of pharmacists in China.

## 1. Background

Pharmacy, a vital component of healthcare systems worldwide, relies on the indispensable role of pharmacists. In recent years, the Chinese pharmaceutical industry has multiplied, with pharmacists playing critical roles in drug discovery, research, and public health initiatives such as combating COVID-19. This rapid expansion expansion has increased responsibilities for Chinese pharmacists, who now manage traditional Chinese medicine (TCM) and Western medicine, ensuring patient safety through accurate prescriptions, drug interactions, and mitigating side effects. These demands, coupled with the long working hours and irregular shifts common in hospital settings, have led to significant professional demands for Chinese pharmacists, which can lead to conflicts between their work and family responsibilities. Balancing these responsibilities with family duties, such as childcare and eldercare, becomes increasingly complex, further contributing to work family conflict. Pharmacists who experience burnout usually have high WFC, lose interest in their work, and exhibit reduced job performance, which can compromise patient safety and healthcare outcomes (Maslach et al., 2001). Psychological flexibility, a fundamental arrangement of cycles, is a powerful tool that assists individuals in managing stressors and engaging in adaptable behaviour that promotes values-driven action and improves job performance (Gloster et al., 2017). PF can enhance individual job performance by lowering stress and feelings of workplace exclusion, thereby enlightening us about its crucial role in the healthcare profession.

## 1.1. Work family conflict (WFC)

Work family conflict occurs when the demands of work and home responsibilities become somewhat conflicting, making it harder to fulfil one duty because of the other (Pattusamy & Jacob, 2016). Smoktunowicz and Cieślak (2017) stated that this conflict can manifest in two directions: family-to-work conflict, where family responsibilities interfere with work tasks, and work-to-family conflict, where job demands negatively impact family responsibilities. The consequences of WFC are multifaceted, affecting individuals, families, and organisations alike. On a personal level, WFC can lead to emotional exhaustion, life dissatisfaction, and mental health issues (Zhang et al., 2012). Families experience marital discord, increased stress in caregiving, and the struggles of managing childcare and eldercare (Dugan, Kusel et al., 2012). For organisations, WFC often results in absenteeism, lower job performance, turnover, and reduced employee commitment (Allen et al., 2000). The potential for burnout in pharmacists, particularly those in hospital and retail environments with irregular shifts, is a pressing concern that necessitates targeted interventions to ensure their ability to perform effectively at work.

## 1.2. Psychological flexibility

Psychological flexibility (PF) is an individual's capacity to fully engage in the present moment, adapt their behaviour based on changing situational demands, and pursue value-based goals (Hayes et al., 2020). PF is not just a coping tool but a trait that reflects one's ability to manage internal processes, such as stress and emotional distress while maintaining goal-directed behaviour. The six key processes of PF are acceptance, cognitive diffusion, present-focussed attention, self-as-context, committed action, and values to work together to enable individuals to navigate complex and stressful situations more effectively (Hayes et al., 2006). For pharmacists, developing psychological flexibility is crucial in managing the intense demands of their profession. For instance, a pharmacist could use acceptance to acknowledge the stress of a busy day, but still focus on the task at hand. Studies have shown that low levels of stress, anxiety, and sadness are linked to higher PF (Hayes et al., 2006). Pharmacists with greater PF are more likely to find innovative ways to maintain stability between work and family accountability, prioritise tasks, and maintain clear boundaries when necessary (Donaldson-Feilder et al., 2011). This ability to adjust and manage competing demands without becoming overwhelmed is directly linked to improved job performance.

Understanding how PF can mitigate the adverse effects of WFC on job performance is vital for healthcare institutions. These institutions play a crucial role in supporting their pharmacists and improving healthcare delivery. High job performance among pharmacists is critical for ensuring patient safety, optimising medication use, and promoting better health outcomes for the general population (Barrick et al., 2001). The present research explores the relationships between WFC, psychological flexibility, and job performance among pharmacists in China. Understanding how these variables interact is crucial for improving pharmacists’ well-being and optimising their job performance, ultimately benefiting patient outcomes. This study provides crucial insights for medical institutions, researchers, and particularly pharmacists in China, highlighting the growing issue of work family conflict within this profession. As work and family demands continue to shift globally, driven by changes in demographics and technological advancements (Kossek, 2016), the rising levels of WFC must be addressed to ensure the well-being of pharmacists and maintain high job performance. Pharmacists experiencing lower WFC, enhanced by psychological flexibility, are better positioned to manage their job responsibilities effectively, leading to improved patient care and reduced errors in medication management. This research can guide interventions and strategies designed to decrease work family conflict and enhance psychological flexibility among pharmacists, benefiting their professional lives and well-being and underlining the urgent need for action to support pharmacists in China.

## 1.3. Objectives and hypotheses

The main objectives of current research are to examine the correlation between work family conflict, psychological flexibility, and job performance, to determine the impact of work family conflict and psychological flexibility on job performance, and to compare levels of these study variables among pharmacists in China. In order to achieve these objectives, the following hypotheses were formulated:

1. There is a significant relationship between work family conflict, psychological flexibility, and job performance of pharmacists.
2. There is a significant impact of work family conflict and psychological flexibility on job performance.
3. The levels of job performance, psychological flexibility, and work family conflict differ among pharmacists according to gender and placement in various regions of China.

## 2. Method

### 2.1. Sample

**Figure 1.**
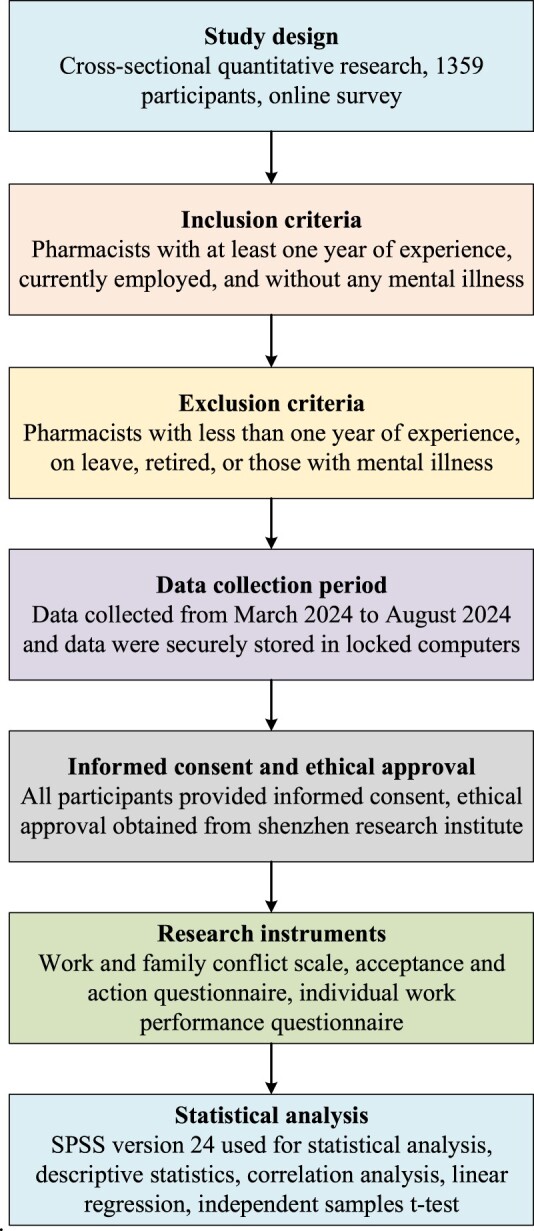
Pipeline of research methodology.

A cross-sectional quantitative research design was used to collect data from both male and female pharmacists across Shenzhen, China. The sample includes a diverse group of pharmacists to ensure comprehensive results. An informed consent was used to inform goals of the study to all participants and given the assurance that any information they submitted would be kept completely private and it will be used only for research purpose. A secure computer system was used to ensure privacy of participant’s data. Data were gathered through an online survey, and data was initially collected from 1380 participants. Data from Twenty-one participants was excluded due to incomplete responses. The selected sample consisted of *N* = 1359 participants (male = 574 and female = 785). The sample was selected from various healthcare settings, such as hospitals, clinics, and retail pharmacies across China. Detailed methodology is shown in Figure 1.

#### 2.1.1. Inclusion criteria

This study selected only pharmacists who are actively employed in hospitals, pharmacies, and health care centres, on the job, without any mental illness, and have at least a year of work experience in pharmacy.

#### 2.1.2. Exclusion criteria

This study excluded pharmacists with less than one year of experience, those with mental illness, those on leave, and retired people. Participants who were aware of this study provided written informed consent.

### 2.2. Research procedure

A structured questionnaire, including a demographic information sheet, was distributed online via email to all participants. The email addresses of the target population were obtained through their respective workplaces to ensure widespread accessibility. The study's objectives were communicated to all participants, and they were assured that all provided information would be kept strictly confidential. All related documents were closely monitored and stored in a protected computer system to maintain security. The data collection took place over six months, from March 2024 to August 2024. An ethical review board of Shenzhen Research Institute, Shenzhen approved this study.

### 2.3. Research instruments

A detailed structured questionnaire including a demographic information sheet (Name, Age, Gender, work experience, job location), the Work and Family Conflict Scale (WAFCS), The Acceptance and Action Questionnaire (AAQ-2), and Individual Work Performance Questionnaire (IWPQ) was sent online to all pharmacists. All questionnaires were in the self-report format.

#### 2.3.1. Work and family conflict scale (WAFCS)

Work Family Conflict Scale (Haslam et al., 2015) has ten items with a seven-point response scale that ranges from one (very strongly disagree) to seven (very strongly agree). Two aspects of conflict are evaluated on this scale: family-work conflict (five items) and work family conflict (five items). The items on each subscale are added to give a total score between 5 and 35 for work family conflict and 5–35 for family-work conflict. Higher scores indicate more conflict. With a Cronbach's alpha of 0.94, the scale was reliable in the current study.

#### 2.3.2. Acceptance and action questionnaire (AAQ-2)

In 2011, Bond and colleagues developed Acceptance and Action Questionnaire (AAQ-2) (Bond et al., 2013) that measures mental adaptability. There are seven items on the scale, and responses are scored on a 7-point scale from never true (1) to very rarely true (2) to seldom true (3) to sometimes true (4) to frequently true (5) to almost always true (6) to always true (7). Add up the responses to each item to determine the AAQ-2's score. A higher level of psychological flexibility is reflected in higher scores. In the current study, the reliability of this scale was = 0.94.

#### 2.3.3. Individual work performance questionnaire (IWPQ)

Individual Work Performance Questionnaire (IWPQ) was initially developed by Koopmans (Koopmans et al., 2012). The current study measured pharmacists’ work performance using an updated version of the IWPQ. There are three subscales for the 18 items on this scale: task performance (items 1–5), contextual performance (items 6–13), and work behaviour that is counterproductive (items 14–18). These subscale scores range from 0 to 4, with higher scores indicating improved work performance. With a Cronbach's alpha of 0.85, the scale's reliability was high for the current study.

### 2.4. Statistical analysis

In this study, SPSS version 24 was utilised for inferential statistical analyses. Descriptive statistics are used to report basic demographic information. Correlation analysis was applied to determine the relationships among work family conflict, psychological flexibility, and job performance. Additionally, linear regression analysis was conducted to evaluate the impact of work family conflict and psychological flexibility on job performance. In order to measure differences in work family conflict, psychological flexibility, and job performance based on gender and job placement areas, an independent samples t-test was utilised. These methods offered a thorough understanding of the connections and outcomes of the research variables.

## 3. Results

### 3.1. Demographic data of participants

This study included 1359 pharmacists from different healthcare settings in China. Most of the staff was 20–30 years old, accounting for 61.1% (*n* = 823) of the sample. Gender distribution was slightly skewed towards females, with a considerable proportion of females 58.0% (*n* = 785). Regarding the area of job placement, 60.5% (*n* = 823) of the participants were from metropolitan areas, whereas 39.5% (*n* = 536) were from rural settings. Regarding years of experience, the majority, 38.5% (*n *= 523), had 5–10 years. Employment status revealed that 60.2% (*n* = 818) were employed full-time, and 39.8% (*n* = 541) were part-time employees. Marital status showed that 62.9% (*n* = 855) of the participants were married. As for the workplace setting, a majority worked in hospitals (66.5%, *n* = 896), followed by healthcare centres (25.4%, *n* = 381) and pharmacies (8.1%, *n* = 109). These detailed demographic insights in Table 1 provide valuable information about the characteristics of the study participants.

**Table 1.** Basic characteristics of participants.

Demographics	Frequency	Percentage (%)
**Age group**		
20–30 years	823	61.1
31–40 years	367	26.6
41–50 years	169	12.3
**Gender**		
Male	574	42.0
Female	785	58.0
**Area of working**		
Metropolitan	823	60.5
Rural	536	39.5
**Year of experience**		
1–5 years	502	37.0
5–10 years	523	38.5
10 + years	334	24.5
**Employment status**		
Full time	818	60.2
Part time	541	39.8
**Marital status**		
Single	322	23.0
Married	855	62.0
Separated	182	15.0
**Workplace**		
Hospital	896	65.9
Pharmacy	82	6.0
Health care centre	381	28.0

### 3.2. Reliability of study variables

Reliability Analysis was performed to determine the internal consistency reliability of psychometric measures, as presented in Table 2. WFC with the mean score of 35.12 and standard deviation of 15.09 showed Cronbach's alpha value of 0.94, suggesting excellent internal consistency. The skewness value was .391, and the kurtosis was –.426, indicating a slightly positive skew and a relatively flat distribution. PF had a mean score of 25.10 and a standard deviation of 12.91 with a Cronbach's alpha of .94. The skewness was .261, and the kurtosis was –1.022, suggesting a distribution that is moderately positively skewed and flatter than a normal distribution. Job performance reported a mean of 40.70 and a standard deviation of 12.61, with Cronbach's alpha of .85, representing good internal consistency. The skewness was –.468, and the kurtosis was .247, reflecting a slight negative skew and a distribution close to normal kurtosis.

**Table 2.** Descriptive statistics and psychometric properties of work family conflict, psychological flexibility and job performance.

Variables	*M*	*SD*	Cronbach's alpha	^a^Skewness	^b^Kurtosis
Work family conflict	35.12	15.09	.94	.391	−.426
Psychological flexibility	25.10	12.91	.94	.261	−1.022
Job performance	40.70	12.61	.85	−.468	.247

Note*: a* = Standard error of skewness* = .130; b *= Standard error of kurtosis* = .260.*

### 3.3. Strength and direction of relationship

Pearson correlations were used to examine the correlation among work family conflict, psychological flexibility, and job performance, as presented in Table 3, revealed that work family conflict has a significant positive relationship with psychological flexibility (*r* = .685, *p* < .001) and job performance (*r* = .181, *p* < .01). Psychological flexibility also shows a significant positive correlation with job performance (*r* = .168, *p* < .01).

**Table 3.** Correlation among work family conflict, psychological flexibility and job performance.

	Variables	1	2	3
1.	Work family conflict	–		
2.	Psychological flexibility	.685***	–	
3.	Job performance	.181**	.168**	–

Note: **p < .01, p*** < .001.

### 3.4. Regression analysis predicting job performance

Regression analysis was exploited to analyse the effects of work family conflict and psychological flexibility on job performance. As presented in Table 4, the work family conflict demonstrated a significant negative impact on job performance among pharmacists. The *R*² value of 0.033 indicates that this predictor variable explains approximately 3% of the variance in job performance. The findings revealed that work family conflict negatively predicts job performance, with a standardised coefficient (Beta) of 0.18 (*p* < .001). This suggests that higher levels of work family conflict are connected with low job performance among the pharmacists in the study. Reducing work family conflict is essential to enhancing the general well-being and productivity of pharmacists.

**Table 4.** Regression coefficient of work family conflict and psychological flexibility on job performance.

Variables	B	Beta	*SE*	*p*
Work family conflict	−.151	−.18***	.044	.001
*R*^2^	.033***			
Psychological flexibility	.164	.168**	.052	.002
*R*^2^	.028**			

Note: **p < .01, p*** < .001.

Regarding the impact of psychological flexibility on job performance among pharmacists, The *R*^2^ value of .028 disclosed that the predictor variable explained a 2.8% variance in the outcome variable. The findings unconcealed that psychological flexibility positively predicted job performance (Beta = .168, *p* < .01).

### 3.5. Difference in job performance, psychological flexibility, and work family conflict among pharmacists regarding area of work and gender

Job performance, psychological flexibility and work family conflict scores of pharmacists working in metropolitan and rural areas were compared through an independence t-test, as shown in Table 5. On average, pharmacists in metropolitan areas have higher job performance (M = 41.40, SD = 13.28) than rural pharmacists. However, the difference between the two groups was not statistically significant (*p* = .657), with a minimal effect size (Cohen’s d = 0.047). This indicates that job performance levels are comparable between metropolitan and rural pharmacists. Psychological flexibility (M = 27.43, SD = 14.57) is higher among Metropolitan area pharmacists, and this difference was statistically significant (*p* = .001) and had a moderate effect size (Cohen’s *d* = 0.365). This suggests that pharmacists in metropolitan areas exhibit greater psychological flexibility than those in rural areas. Comparatively, work family conflict is higher among metropolitan area pharmacists (M = 37.03, SD = 17.22) than among pharmacists in rural areas. This difference was statistically significant (*p* = .018) with a diminutive effect size (Cohen’s *d* = 0.254), indicating that those in metropolitan areas experience more work family conflict than their rural counterparts.

**Table 5.** *t-*test for comparison of job performance, psychological flexibility and work family conflict between pharmacists working in metropolitan and rural areas.

Variables	Metropolitan		Rural			
	*M*	*SD*	*M*	*SD*	*p*	Cohen’s *d*
Job performance	41.40	13.28	40.0	11.94	.657	0.047
Psychological flexibility	27.43	14.57	22.78	10.55	.001	0.365
Work family conflict	37.03	17.22	33.22	12.37	.018	0.254

Regarding gender, the mean difference for males (M = 21.40, SD = 3.70) is higher than for females (M = 20.9, SD = 2.70) for job performance. The mean difference for males (M = 45.06, SD = 8.71) is higher than for females (M = 43.10, SD = 7.00) for Psychological flexibility. The mean difference for males (M = 60.6, SD = 9.85) is lower than for females. (M = 62.17, SD = 11.45) All these differences are insignificant for work family conflict, as shown in Table 6.

**Table 6.** *t*-test for comparison of job performance, psychological flexibility and work family conflict between male and female pharmacists.

Variables	Male		Female			
	*M*	SD	*M*	*SD*	*p*	Cohen’s *d*
Job performance	21.40	3.70	20.9	2.70	.297	0.34
Psychological flexibility	45.06	8.71	43.10	7.00	.083	0.31
Work family conflict	60.6	9.85	62.17	11.45	.339	0.29

## 4. Discussion

The primary goal of the recent research was to explore the impact of the Work Family Conflict and Psychological Flexibility on the Job Performance of Chinese Pharmacists. The other purposes of our research were to determine the level of work family conflict, psychological flexibility, and job performance among male and female pharmacists in different regions of China. The findings provide important insights into these dynamics, revealing significant correlations and the influence of Work Family Conflict and Psychological Flexibility on Job Performance.

The impact of Work Family Conflict on job performance was analysed, and our results align with previous studies, providing a robust foundation for our findings. We found that Work Family Conflict adversely influences job performance among pharmacists, a conclusion that is consistent with the research of Majekodunmi et al. (2017). Their study also revealed a strong inverse correlation between job interference and a worker's ability to fulfil their tasks at the workplace. Our results indicate that workers significantly affected by work family conflict often feel worn out and require more time or energy, making it challenging to concentrate on their work. Furthermore, Nohe and colleagues (2014) suggested that family obligations may clash with professional duties, leading to negative outcomes for workers. The interference between family and work negatively and significantly affects workers’ ability to perform their tasks at work. Furthermore, these findings also align with earlier research conducted by Ahmad (2013), Ling and Jane (2014) and Karakas and Sahin (2017), who discovered a substantial correlation between Work family Conflict and Job Performance.

The Psychological Flexibility effect on Job Performance among Chinese pharmacists was also measured. The findings indicated that Psychological Flexibility significantly predicted job performance. The recent study by Yang et al. (2021) supports our findings that psychological flexibility positively impacts job tasks. According to Gloster et al. (2017), psychological flexibility can manage stressors and pursue values through adaptive behaviour. The individual with elevated psychological flexibility or positive perception can overcome work-life challenges. Effective use of functional coping strategies can boost work performance and job satisfaction (Ochoa Pacheco et al., 2023).

The level of job performance, psychological flexibility, and work family conflict between pharmacists in metropolitan and rural areas was a key focus of our analysis. We discovered that pharmacists in metropolitan areas exhibit a higher level of psychological flexibility in managing work family conflicts than their rural counterparts. This adaptability was particularly evident in a comparative study of urban and rural employees in China (Zhou et al., 2019). Another study also found that psychological flexibility positively correlates with job performance in urban and rural employees, but through different mechanisms (Wu et al., 2018). In urban settings, this adaptability is a crucial tool for employees to navigate complex job tasks, adapt to fast-paced changes, and manage work-life balance. In rural areas, flexibility is often used to manage family and community roles alongside work demands, especially in environments with fluctuating agricultural or seasonal labour demands (Mao et al., 2020). This highlights the resilience of rural pharmacists in the face of unique challenges. Previous research found significant work family conflict among urban employees compared to rural employees due to rigid work schedules and long working hours (Wu et al., 2018). Wang et al. (2017) conducted a study showing the adverse effect of work family conflict on job performance for both urban and rural areas employees. However, urban employees may have higher job performance due to better family support, working conditions and training programmes. A recent study in China showed that urban employees perform better than rural area employees on complex tasks (Jiang, Chen et al., 2020). Urban employees in China often benefit from better access to modern technology and digital tools, which improves their job performance. According to Gao et al. (2020), utilising technology in the workplace significantly boosts productivity in urban areas.

A comparison of job performance, psychological flexibility, and work family conflicts between male and female pharmacists was also conducted. Results concluded that male pharmacists have higher psychological flexibility and job performance than female pharmacists, while work family conflict is higher among females than male pharmacists in Shenzhen, China. Morley et al. (2016) explored how psychological flexibility supports stress management in male and female employees. They reported that female employees demonstrated higher psychological flexibility when managing emotional stress and role conflicts. In comparison, male employees displayed more flexibility in dealing with work-related tasks and goal-setting. Michel et al. (2011) stated that work family conflict indicated job performance decline for women, while for males, work family conflict caused distress in people but had less of an immediate impact on job performance. Si et al. (2015) explored how work family conflict impacted job performance among male and female employees in China. Their research demonstrated that women's job performance was more negatively impacted by family responsibilities.

## 4.1. Practical implications

**Figure 2.**
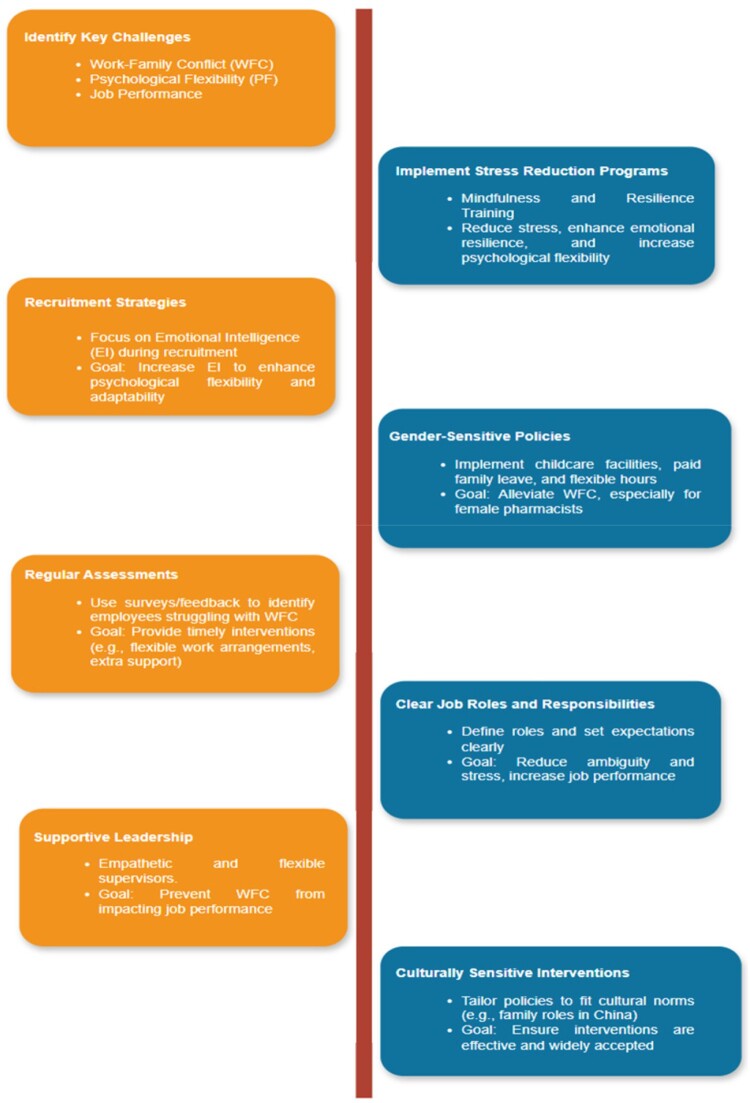
Flow chart for practical implications for reducing work family conflict and improving job performance.

The findings of this study suggest several practical strategies to address WFC and enhance job performance among pharmacists as shown in Figure 2. Hospital management can implement stress reduction and mindfulness programmes to help pharmacists cope with work-related stress and build psychological flexibility, improving their job performance. Additionally, focussing on emotional intelligence (EI) during recruitment can increase psychological flexibility, as pharmacists with higher EI tend to manage stress and work-life balance more effectively. Gender-sensitive policies are essential, as traditional gender roles contribute to higher WFC among female pharmacists. Providing on-site childcare, paid family leave, and flexible working hours can help alleviate the strain on female pharmacists, reducing WFC and boosting productivity. Regular assessments of WFC and job performance through surveys can help identify employees facing challenges, allowing for timely interventions such as flexible work arrangements. Clear job roles and responsibilities, along with supportive leadership, can also mitigate WFC. Supervisors who offer empathy and flexibility can prevent WFC from negatively impacting job performance. Lastly, interventions should be culturally sensitive, particularly in the Chinese context, where societal expectations around gender and family play a significant role. By aligning policies with cultural norms while promoting work-life integration, healthcare organisations can foster a more supportive environment.

## 4.2. Limitations and future research

Cross-sectional design selected in the recent research. The cross-sectional design limits the causal conclusion. Cross-sectional designs can miss temporal patterns or trends by failing to record fluctuations in these variables. To investigate how work family conflict, psychological adaptability, and job performance evolve over time, longitudinal research is required. Other sociocultural factors such as education, age, professional experience and social class can also be examined as moderators. The study of Chinese pharmacists takes place in a distinct area, so findings might not apply to pharmacists working in other areas. The experience of WFC and psychological flexibility may be influenced by the hierarchical and collectivist aspects of Chinese society, which may restrict the findings’ applicability to individualistic or Western societies. Although psychological flexibility has a positive effect, not every person is naturally flexible. Therefore, other personal characteristics such as emotional intelligence, self-efficacy, resilience and social support must be considered in future research. Future studies should focus on introducing training programmes to expand psychological flexibility and reduce the stress level induced by work family conflict. As the healthcare system has changed since COVID-19, future research should consider telemedicine, working from home, and digital health factors.

## 5. Conclusion

In conclusion, this study highlights the significant positive impact of psychological flexibility on job performance and the negative impact of work family conflict on job performance among pharmacists in China. This study also highlights higher psychological flexibility, work family conflict, and job performance among pharmacists in metropolitan areas than among rural pharmacists. Results also concluded that male pharmacists have higher psychological flexibility and job performance than female pharmacists, while work family conflict is higher among females than male pharmacists in China. These results highlighted the necessity of efficient treatments to deal with work family conflict. It can have a negative impact on hospital operations and patient care by causing lower work satisfaction, more absenteeism, and high employee turnover rates. Therefore, keeping highly productive, healthy, and encouraging work environments requires putting comprehensive solutions to manage and prevent work family conflict into practice. These findings also demonstrate the importance of supporting psychological flexibility in the workplace to enhance worker productivity and well-being. Better performance results can result from interventions that enhance pharmacists’ capacity to adjust to changing conditions, deal with work family conflicts, and stay focussed on their duties. In order to improve job happiness and performance, employers should also consider putting in place supportive policies and work environments that lessen work family friction, like flexible work schedules or mental health resources.

## Data Availability

On demand.
